# Predictors of Nicotine Replacement Therapy Adherence: Mixed-Methods Research With a Convergent Parallel Design

**DOI:** 10.1093/abm/kaae006

**Published:** 2024-02-24

**Authors:** Sun S Kim, Anyah Prasad, Manan M Nayak, Hua Chen, Chaowalit Srisoem, Rosanna F DeMarco, Peter Castaldi, Mary E Cooley

**Affiliations:** Department of Nursing, Manning College of Nursing and Health Sciences, University of Massachusetts Boston, Boston, MA, USA; Department of Gerontology, Manning College of Nursing and Health Sciences, University of Massachusetts Boston, Boston, MA, USA; Department of Psychosocial Oncology and Palliative Care, The Phyllis F. Cantor Center Research in Nursing and Patient Care Services, Dana-Farber Cancer Institute, Boston, MA, USA; Department of Nursing, Manning College of Nursing and Health Sciences, University of Massachusetts Boston, Boston, MA, USA; Department of Nursing, Manning College of Nursing and Health Sciences, University of Massachusetts Boston, Boston, MA, USA; Department of Nursing, Manning College of Nursing and Health Sciences, University of Massachusetts Boston, Boston, MA, USA; Channing Division of Network Medicine, Department of Medicine, Brigham and Women’s Hospital, Harvard Medical School, Boston, MA, USA; Phyllis F. Cantor Center, Research in Nursing and Patient Care Services, Dana-Farber Cancer Institute, Boston, MA, USA

**Keywords:** Nicotine replacement therapy, Adherence, Attitudes, Smoking cessation, Mixed-methods research

## Abstract

**Background:**

Few studies have examined the effect of baseline attitudes toward nicotine replacement therapy (NRT) on its actual adherence in a smoking cessation intervention.

**Purpose:**

This study (i) examined the predictability of baseline variables (quantitative data) on NRT adherence and (ii) explored the congruence of participants’ statements about NRT products (qualitative data) during counseling sessions with their baseline attitudes.

**Methods:**

This is a mixed-methods research study using a convergent parallel design. Participants included 74 individuals in the treatment group who received behavioral counseling and combination NRT. A Poisson regression analysis was performed to identify baseline variables predicting NRT adherence. Thematic analysis was completed with a subset of participants (*n* = 38) who varied in NRT attitude scores and adherence. A joint display was created to integrate quantitative and qualitative data and discover convergence.

**Results:**

Approximately 59% of the participants (41/74) used NRT continuously for ≥5 weeks. Having negative attitudes toward NRT and depressive symptoms predicted NRT adherence even after controlling for education and anxiety symptoms. Thematic analysis revealed that NRT adherence is a learning process that consists of the following three distinctive but interrelated phases: (i) information needs, (ii) comprehensive readiness, and (iii) experiential learning. Of the 38 participants, 34 (89.5%) showed convergence between baseline attitude scores and statements about NRT made during counseling sessions.

**Conclusions:**

Individuals who have negative attitudes toward NRT are less likely to use the products in a smoking cessation intervention. Counselors should assess attitudes toward NRT at baseline and address them proactively during counseling sessions.

## Teaser

Individuals who smoke and have negative attitudes toward nicotine patches, lozenges, and gum are less likely to use the products during their cessation attempts.

## Graphical Abstract

**Graphical Abstract:**
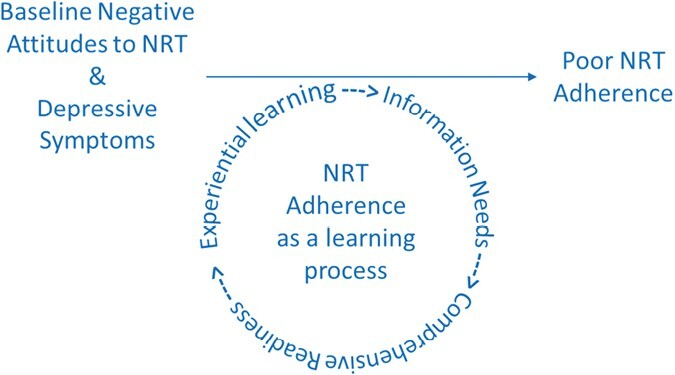

## Introduction

Smoking is the number one leading cause of preventable deaths in the U.S. killing more than 480,000 people every year, including 41,000 people who die from exposure to secondhand smoke [1]. In 2021, 11.5% (28.3 million) of U.S. adults 18 or older currently smoked cigarettes [2], and more than half (60.2%) of these people reportedly attempted to quit smoking within the past year [3]. Evidence supports that a combination of pharmacotherapy and behavioral counseling can increase the likelihood of successful smoking cessation [4, 5]. While there are several FDA-approved medications for smoking cessation, nicotine replacement therapy (NRT) is comparatively effective and easily accessible over the counter. Combination NRT, such as a dual-usage of nicotine patches and nicotine lozenges or nicotine gum, has been found to be more effective than the usage of a single-agent NRT because the combination supplies a steady release of nicotine in a 24-hr period while providing a fast-acting product to address nicotine cravings [6–8]. However, a recent study identified that only 10% of individuals who tried to quit smoking used NRT as a cessation aid [9].

NRT adherence can double the success of smoking cessation [7, 10]. Mersha et al. developed a framework to illustrate composite factors associated with NRT adherence using the Capability, Opportunity, Motivation, and Behavior (COM-B) model [11]. They identified 31 factors from a systematic review of 26 studies. They mapped the factors into the six elements of the COM-B model: psychological capability (forgetfulness and education), physical capability (level of nicotine dependence and withdrawal symptoms), reflective motivation (perception about NRT and quitting), automatic motivation (alcohol use, stress, and depression), physical opportunity (cost), and social opportunity (social support) [11]. The COM-B model was used to guide the quantitative part of the present study and the selection of variables that might affect adherence to NRT. According to Mersha et al., reflective motivation, defined as positive and negative perceptions and beliefs, was the most prominent element associated with NRT adherence [11]. Examples provided for the construct (perceiving NRT as noneffective, believing NRT is safe, believing NRT is easy to use, etc.) are identical to several question items in the 12-item Attitudes toward Nicotine Replacement Therapy (ANRT-12) scale developed by Etter and Perneger [12]. The COM-B model also includes depression, anxiety, and social support as factors associated with NRT adherence.

Numerous studies have been conducted to identify demographics and psychosocial variables associated with NRT adherence. Males, older people, and more educated people were more adherent to the medications than their respective counterparts [13, 14]. Having good social support [15] and believing NRT was safe and easy to use [16] were also found to increase the odds of being adherent. In contrast, low socioeconomic status [17] and having depressive or anxiety symptoms [15, 18] were found to reduce adherence. Findings are mixed regarding the relationship between nicotine dependence and NRT adherence. Some studies found a positive relationship [15, 16, 19–21], whereas others reported an inverse relationship [22–24]. Nevertheless, few published studies tested the effect of baseline NRT attitudes on actual adherence during a smoking cessation intervention. This study extends the literature by using a mixed-methods approach to examine if baseline NRT attitudes, sociodemographic factors, and other psychological variables such as depressive and anxiety symptoms would predict NRT adherence (quantitative data) and to explore whether participants’ statements (qualitative data) during counseling sessions would convey concordant messages with their baseline attitude scores.

The following hypotheses were proposed as part of the quantitative study:

Individuals who had more positive attitudes toward NRT at baseline would be more likely to adhere to NRT during the smoking cessation intervention.
Individuals who had more negative attitudes toward NRT at baseline would be less likely to adhere to NRT during the smoking cessation intervention.
Individuals who had more depressive symptoms at baseline would be less likely to adhere to NRT during the smoking cessation intervention.
Individuals who had more anxiety symptoms at baseline would be less likely to adhere to NRT during the smoking cessation intervention.

## Method

### Research Design

This study is mixed-method research using a convergent parallel design. This design was selected to illustrate the convergence of qualitative data with quantitative data regarding attitudes toward NRT. The study is a secondary data analysis of a two-arm randomized controlled trial that tested the efficacy of a digitized lung health intervention conducted at an academic cancer institute. The treatment group received eight weekly individualized counseling sessions over Zoom for smoking cessation and education about low-dose computed tomography (CT) scan lung cancer screening, and the control group was referred to the Quitline after brief smoking cessation advice over the telephone. Therefore, those delivering the intervention and participants receiving the intervention were not blinded to group assignments. The quantitative data were assessed at baseline and during eight weekly counseling sessions. The qualitative data were participants’ statements about NRT extracted from counseling sessions. We used participants’ statements from the treatment arm only because participants in the control arm did not receive individualized counseling sessions from the study team. The study was approved by the institutional review board of an academic cancer institute, and informed consent was obtained from all participants prior to baseline data collection. Detailed descriptions of the parent study have been reported elsewhere [25].

### Participants

Participants were community-dwelling residents recruited remotely across the nation via Facebook advertisements. Inclusion criteria for study participation were individuals who: (i) were eligible for the low-dose CT scan (55–77 years of age, 30-pack-year smoking history, and Eastern Cooperative Oncology Group performance status ≤1), (ii) had smoked at least 5 cigarettes per day for the past 30 days, (iii) owned a mobile phone or another electronic device to access a video call application, (iv) had a primary care provider, (v) had active health insurance, and (vi) did not have the low-dose CT scan lung cancer screening before. The exclusion criterion was any hospitalization due to serious mental illness during the past 6 months. We launched Facebook advertisements linking to a Lung Health Study website on January 11, 2021, and we recruited participants between January 2021 and June 2022.

### Intervention

Detailed descriptions of the intervention and counselor training are described in the study protocol published elsewhere [25]. The first author was the lead counselor who had more than 15 years of experience in smoking cessation counseling, and the other two were doctoral students trained by the first author and received 4 days of intensive training from an accredited tobacco treatment training program. In the first session, counselors assessed participants’ willingness to use transdermal nicotine patches and their preference between the usage of nicotine gum and lozenges for a short-acting NRT product. Participants were mailed the first 4-week supply of patches combined with gum or lozenges depending on their preference. They also received information materials instructing them on how to use the medications and manage common side effects. The dosages of NRT were adjusted according to the number of cigarettes smoked per day or time to the first cigarette after waking. At the beginning of each session, counselors asked participants how many nicotine patches and gum or lozenges they had used since the last session. During the counseling session, counselors explained the underlying treatment mechanism of NRT and addressed any side effect concerns in real time. They reassured participants that such side effects were manageable and that nicotine patches were safe even if they wore them while smoking cigarettes. We mailed the second 4-week supply of the medications 3 weeks after the first package and after counselors confirmed participants’ self-report affirming adherence to the medications. Although we planned to do a salivary cotinine test for the validation of self-reported abstinence at the end of the intervention, we could not do so because many participants reported using NRT during the prior 7 days.

### Data Collection

Participants self-administered baseline questionnaires at home that included sociodemographic information and tobacco-related variables (smoking and quitting behaviors and attitudes toward NRT), and psychosocial variables (depressive and anxiety symptoms). The assessment was done on a HIPAA compliant web-based Research Electronic Data Capture (REDCap). Those who had difficulty accessing the REDCap through the Internet completed the questionnaires via mailed surveys or by telephone. Adherence to NRT and smoking status were assessed weekly at the beginning of each counseling session. Qualitative data were participants’ statements about NRT during counseling sessions, which were audiotaped. First, we selected participants stratified by three counselors who provided the intervention. Second, we selected participants whose NRT attitude scores varied from the mean. Third, we selected participants whose NRT use varied by the number of weeks. We then selected participants who differed in smoking cessation outcome and gender. As a result, 38 participants were selected. The first author listened to the recordings of selected participants and then transcribed the sections where participants talked about NRT. A second person verified the transcripts while listening to the recordings. The session extracted most from was either the fifth or the sixth session when participants had been abstinent for about 1–2 weeks. The first session also had many statements related to NRT in which counselors asked participants their prior experiences of NRT use and preference between nicotine gum and lozenges. Several participants also offered their overall experiences with NRT at the eighth session.

### Measures

#### Sociodemographic Questionnaire

We collected information on age, sex assigned at birth, race and ethnicity, marital status, employment status, and education level.

#### Smoking and Quitting Questionnaire

We gathered information about the age at smoking onset, the number of cigarettes smoked per day on average, and past year quit attempts at which abstinence lasted more than 24 hr.

#### The Heaviness of Smoking Index

Nicotine dependence was assessed using the Heaviness of Smoking Index composed of two questions: time to first cigarette of the day (TTFC) and the number of cigarettes smoked per day on average (CPD) [26]. The codes of TTFC are 0: 61+ min, 1: 31–60 min, 2: 6–30 min, and 3: ≤5 min [26]. The codes for CPD are 0: 1–10, 1: 11–20, 2: 21–30, and 3: 31+. The scale score is the sum of the two scores with a range of 0–6. The scale was found to be a valid and reliable measure of nicotine dependence [27].

#### ANRT-12 scale

This scale has two subscales that measure the perception of the advantages (positive attitudes) of NRT (eight items) and the drawbacks (negative attitudes) of NRT (four items) [12]. The measure is a 5-point Likert-type scale ranging from “strongly disagree” to “fully agree” that are coded “0” and “4,” respectively. Examples of positive attitudes are “*These products help people to feel less irritable when they quit smoking*” and “*These products help people to feel less depressed when they quit smoking.*” Negative attitudes include statements such as “*I am concerned about the side effects of these products*” and “*I am wary of these products*.” A sixth response option labeled “don’t know” was coded as a missing value following the scale developers’ suggestion. The two subscales use the mean of the eight-item scores for advantages and the mean of the four-item scores for drawbacks. The score ranges from 0 to 4, and the higher the score is, the more positive (advantages) or negative (drawbacks) attitudes an individual has. Cronbach’s alpha was 0.87 for the advantage subscale and was 0.68 for the drawback subscale. Etter and Perneger reported that the scale scores were associated with the intention to use NRT and the number of NRT-use days, suggesting the construct validity of the measure [12].

#### Alcohol Use Disorders Identification Test-Consumption

This was assessed using three items of the 10-item scale; each scored on a 5-point scale from “0” to “4.” The scale score is the sum of the three-item scores (0–12), with a score of 0 reflecting no alcohol use [28]. Individuals with scores of ≥4 (male sex at birth) or ≥3 (female sex at birth) were categorized as having “high-risk alcohol use.” Cronbach’s alpha was 0.70 in this study.

#### Patient Health Questionnaire 4-item scale

The scale is a four-item questionnaire answered on a 4-point Likert-type scale. It consists of the core symptoms and signs of anxiety and depression by combining the first two items of the Generalized Anxiety Disorder-7 scale for anxiety and the first two items of the Patient Health Questionnaire 4-item (PHQ-8) for depression [29]. A total score of ≥3 for the first two questions suggests anxiety, and a total score of ≥3 for the last two questions suggests depression. Cronbach’s alpha was 0.86 in this study.

#### Adherence to counseling sessions

Adherence to counseling sessions was assessed by the number of sessions attended. Those who had attended all eight weekly sessions without missing one were coded as adhering to the counseling session.

#### NRT adherence

This was defined as the number of weeks during which participants had used any NRT products as recommended. Counselors assessed daily use of NRT using the timeline follow-back scale [30] during weekly counseling sessions.

#### Smoking abstinence

Participants were asked about having smoked any cigarettes, even a puff, for the past 7 days at the eighth counseling session. Those who answered “no” to the question were recorded as being abstinent, while those who answered “yes” were then assessed using the timeline follow-back scale [30]. Individuals who were missing or dropped out of the study even before receiving any part of the intervention were all treated as smoking at the baseline level.

### Data Analysis

#### Quantitative analysis

To see the sample size calculation, please refer to the study protocol [25]. Data were analyzed using STATA Statistical Software 15 (Stata Corp, 2017). Descriptive statistics of demographic information and other key variables were performed. Correlations of key study variables were calculated. Pearson’s correlation coefficients were run when both variables were continuous measures. Otherwise, Spearman’s rank coefficients were run. Due to the non-normal distribution of weeks of NRT use, a Poisson regression analysis was performed. We selected sociodemographic factors (gender, age, and education) as covariates that have been found to be significant correlates of NRT adherence. Incident rate ratios (IRRs) with 95% confidence intervals (CIs) were estimated for predictors. The proportion of missing data was around 3.5%, and the average value of the responses was used to handle missing data if the missingness was less than 25% of the total responses. Cessation outcome was assessed at the eighth counseling session using an intention-to-treat analysis in which all participants assigned to the treatment group were included. A *p*-value ≤.05 was considered statistically significant.

#### Qualitative analysis

The transcripts of 38 participants were imported to NVivo 12 (QSR International, Melbourne, Australia) and analyzed by three coders using the thematic analytic procedures as outlined by Braun and Clarke [31, 32]. The coding was guided by the following research questions: (i) What did participants report about NRT? and (ii) how did the reports vary by NRT adherence? The three coders independently generated themes from the repetitive codes that they created. They then compared the themes and discussed to reach a consensus for different themes. The agreed themes were then reviewed by the remaining research team members. The three coders and three additional research team members discussed the emerging themes until they reached agreement. They renamed some of the themes, grouped them into major categories, and found an overarching theme capturing participants’ adherence to NRT. The first author then randomly listened to audiotaped counseling sessions of 10 participants whose counseling sessions were not transcribed. There was no new information about NRT adherence, suggesting data saturation.

#### Integration of quantitative and qualitative data

We created a joint display to compare the scores of baseline attitudes toward NRT with statements from counseling sessions. Comparing the two datasets strengthened the validity of our findings through the congruence of quantitative and qualitative results (examining concordance and consistency of results) and complementarity (using one dataset to enrich or explain the other) [33]. We also presented discordant cases whose statements did not reflect their baseline attitudes scores. We explored what might have affected the discrepancy between the quantitative and qualitative data.

## Results

Seventy-five participants were initially assigned to the treatment group; however, one was excluded from the study after the person disclosed vaping electronic cigarettes only during the first counseling session. The descriptive statistics of baseline data (*N* = 74), including sociodemographic and key study variables, are shown in Table 1. Participants’ mean age was 62.0 (standard deviation = 4.8) years. They were predominantly non-Hispanic Whites (89.2%) and females (79.7%). Approximately 42% of them were separated, divorced, or widowed in marital status. Nearly 80% of them had higher than secondary education. Individuals reporting “don’t know” on more than one-third of either the advantages (positive attitudes) or the drawbacks (negative attitudes) subscales of the ANRT-12 were excluded from the quantitative analysis. Therefore, the final samples for the two subscales were 65 and 66, respectively. Participants were more likely to endorse positive attitudes toward NRT than negative attitudes. Only five participants endorsed more negative attitudes than positive attitudes that were assessed at baseline. No significant correlation (*r* = −.10, *ns*, Supplementary Table 1) was observed between the two attitude scores.

**Table 1:** Baseline Characteristics (*N* = 74)

Variable	Frequency (%)/mean ± *SD*^a^
Age (years, range 55–76)	62.03 ± 4.76
Gender	
Male	15 (20.27)
Female	59 (79.73)
Races/ethnicity	
Non-Hispanic Black	3 (4.05)
Non-Hispanic White	66 (89.19)
Hispanic White or Black	5 (6.76)
Marital status	
Single or never married	15 (20.27)
Married or living with a partner	28 (37.84)
Separated, divorced, or widowed	31 (41.89)
Employment status	
Working fulltime/parttime	35 (47.30)
Retired	19 (25.67)
Disability/unemployed	20 (27.03)
Educational level	
≤High school or GED^b^	21 (28.38)
All others	53 (71.62)
Age at smoking onset	16.70 ± 3.67
Number of cigarettes per day	20.88 ± 8.86
Past-year 24-hr abstinence (*n* = 72)	44 (61.11)
Nicotine dependence^c^ (0–6)	3.46 ± 1.28
Positive attitudes toward NRT^d^ (1–5, *n* = 65)	2.81 ± 0.70
Negative attitudes toward NRT (1–5, *n* = 66)	1.24 ± 0.82
Alcohol consumption (AUDIT-C^e^)	2.26 ± 2.32
Females (*n* = 59) ≥3	21 (35.59)
Males (*n* = 15) ≥4	5 (33.33)
Depression (*n* = 73, PHQ-2^f^) ≥3	19 (26.03)
Anxiety (GAD-2^g^) ≥3	18 (24.32)

^a^Standard Deviation.

^b^General Educational Development.

^c^The Heaviness of Smoking Index.

^d^Nicotine Replacement Therapy.

^e^Alcohol Use Disorders Identification Test-Consumption.

^f^Patient Health Questionnaire.

^g^Generalized Anxiety Disorder.

### Quantitative Results

Four participants (4/74, 5.4%) dropped out of the study before receiving any counseling sessions. Forty-seven of the remaining participants (47/70, 67.1%) attended all eight weekly counseling sessions, and 41 (58.6%) reported having used NRT products daily for 5 or more consecutive weeks. Among those who adhered, 18 (18/41, 43.9%) used combined NRT, whereas the remaining 23 used a single nicotine product, mostly patches. Based on the intention-to-treat analysis, 28 (28/74, 37.8%) reported that they had not smoked even a single puff during the past 7 days at the eighth counseling intervention. The odds of quitting smoking were much higher (odds ratio = 10.2, 95% CI = 3.0, 34.5) for those who had used NRT for at least 5 consecutive weeks.

A multivariable Poisson regression analysis revealed that negative attitudes toward NRT (IRR = 0.77, 95% CI = 0.66, 0.90) and depressive symptoms (IRR = 0.91, 95% CI = 0.83, 1.00) assessed at baseline were significant predictors of NRT adherence (Table 2). Gender, age, and positive attitudes toward NRT were not significant in the univariable analysis. Therefore, only the four variables, education, negative attitudes toward NRT, depressive symptoms, and anxiety symptoms, were included in the multivariable analysis. Education and anxiety symptoms were no longer significant when negative attitudes toward NRT and depressive symptoms were held constant. As a participant’s negative attitude score increased by one point on the 5-point Likert scale of the ANRT-12, the person’s rate ratio for NRT adherence decreased by a factor of 0.76. Similarly, as a participant’s depressive symptom score increased by one point on the 4-point Likert-type scale of the PHQ-4, the person’s rate ratio for NRT adherence decreased by a factor of 0.89.

**Table 2:** Poisson Regression Analyses of Baseline Variables Predicting Adherence to Nicotine Replacement Therapy

NRT adherence	Univariable analysis (*n* = 65)						Multivariable analysis (*n* = 65)					
	IRR	SE	*z*	*p* > |*z*|	95% CI		IRR	SE	*z*	*p* > |*z*|	95% CI	
Gender (Reference = female)	1.19	1.65	1.28	.200	0.91	1.57						
Age	1.01	0.01	1.19	.236	0.99	1.04						
Education (Reference = ≤12 years or GED)	1.36	0.19	2.18	**.029**	1.03	1.78	1.11	0.16	0.75	.452	0.84	1.47
Positive attitudes toward NRT	1.18	0.11	1.79	.074	0.98	1.42						
Negative attitudes toward NRT	0.77	0.06	−3.42	**.001**	0.66	0.89	0.77	0.06	−3.26	**.001**	0.66	0.90
Depressive symptoms	0.89	0.03	−3.17	**.002**	0.82	0.96	0.91	0.04	−2.00	**.046**	0.83	1.00
Anxiety symptoms	0.92	0.03	−2.36	**.018**	0.85	0.99	0.98	0.04	−0.46	.648	0.90	1.07

*CI* confident interval; *GED* General Educational Development; *IRR* incidence rate ratio; *NRT* nicotine replacement therapy; *SE* standard error; *Z z* score. Bold values represent statistically significant results.

### Qualitative Results

**Fig. 1.**
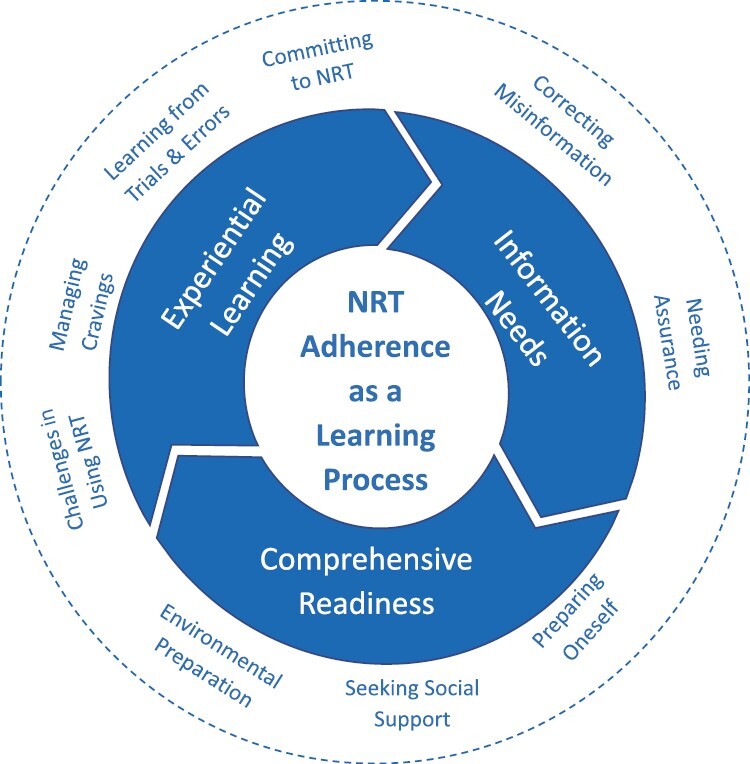
The process of adherence to nicotine replacement therapy.

A total of 115 codes and 15 themes were identified from the statements of 38 participants. The rate of agreement among the three coders was 81.7%. The themes are presented with example quotes in Table 3. The themes were further grouped into three major categories: (i) information needs, (ii) comprehensive readiness, and (iii) experiential learning. An overarching theme, “*NRT adherence is a learning process*,” emerged from the three categories, which is depicted as a wheel of NRT adherence (Fig. 1). The three categories represent distinctive but interrelated phases of the learning process. By going through the process, participants correct misinformation about NRT, get assured of the effects and safety of the medications, prepare themselves mentally and the surrounding social and physical environments before use, gain new knowledge from experiential learning, and eventually become committed to the treatment.

**Table 3:** Themes, Major Themes, and Quotes From Qualitative Data (*n* = 38)

Major themes	Themes	Gender/age	Exemplar quotes
Information needs	Needing assurance	F/62	• Should I leave it (a patch) on while taking a shower. I was wondering whether I should use each separately or use them both (patches and lozenges).
		F/59	• Before I do anything, I want to ask you.
		F/76	• I want to ask you about this. I haven’t tried out one yet, the patches. Do you put it on your arm?
		F/65	• Can I still smoke when I have a patch on?
	Correcting misinformation	F/62	• I don’t wear the patch all the time when I smoke. I am not sure if I can smoke when I put the patch on.
		M/63	• I am upset. I think I was putting much more nicotine by putting 21 mg patches and chewing 4 mg gum. I was doing the 21 mg patch right? And chewing the gum. That is way too much. No way, too much.
Comprehensive readiness	Preparing mentally	F/61	• It’s all mental and psychological. I had to really get the right spirit of mind.
		F/64	• Why don’t we go mentally, so I can prepare myself for this, starting on Sunday… I just want to prepare mentally and dedicate myself to the effort on that day when I start using these (patches and lozenges).
		F/56	• So, having a whole plan, I think it was vital to be successful.
		F/64	• I stepped down to 7 mg. I am on like a third day. Because you told me that, so, I was a little bit prepared for that.
	Seeking social support	F/61	• I told everybody I knew that I was quitting.
		M/60	• Yeah, I let everybody know. I gave everybody heads up that I was quitting.
		M/55	• Over the weekend, we had a party with family friends in the neighborhood down there. I announced that I was quitting smoking.
	Preparing environment	F/66	• I knew I was going on a vacation. I was visiting my friend who lives in a no smoking household. I slapped the patch on my arm. I never thought about cigarettes for the entire 5 days.
		F/64	• Tomorrow, I have three events planned. So, I make psychological and social changes. In that respect, even though I don’t anticipate that I will be smoking with any of these things on that day. I don’t want to be smoking and use patches and lozenges.
Experiential learning	Challenges in using NRT	F/61	• I forgot to put the patch on yesterday. I was on my way to work. I realized I forgot to put the patch on.
		M/60 F/64	• If I have it (a patch) on the arm, muscle flexions make it easily get off.
	Managing cravings	M/56	• I was sweating too much. So, the patch came off.
		F56	• This week after changing to lower dose, it (smoking urge) actually happened in my head So, now I am getting better at dealing with that craving.
		F/62	• You know what? The longer I do the right thing, it easier becomes. I am using both, I got patches and candies (lozenges) … I have been using candies which help me hold up.
		M/56	• I feel a little different after changing [patches] from 21 mg to 14 mg because I feel like the craving is going up. So, it’s a little bit tougher, but I have been trying to keep myself busy, or I try to take a nap. So, I would be less awake and have less chance to crave. I’m happy with the progress.
	Learning from trial and error	F/63	• I wanted to smoke but didn’t want to smoke because I wasn’t feeling comfortable. So, it (wearing patches) did help me to stop smoking for a while. So, that’s helpful. So, that was a, you know, learning experience.
		F/62	• I realized I needed to put that patch on every day. I stopped using patches after relapsing. Well, I didn’t wear the patch at the times, that’s the most big problem for me. On Saturday, I wore a dress, and I didn’t put the patch. I was like I can do this [not smoking] but I could not.
	Committing to NRT	M/66	• I just, just stick with it and get through the day. It has been 21 days.
		F/58	• I am using it (a patch) every day. I think it does work. I don’t know if it’s just my imagination. It’s just what I got to do. As soon as I get up, I switch it over.
		M/60	• I am going to complete the whole cycle. I won’t take any chances.
		M/56	• I still have been using the patches. I’m trying to stick with my schedule.

*NRT* nicotine replacement therapy.

#### Information needs

Nearly all participants reported that they had prior experience of using nicotine patches and gum, whereas only a few reported any experience with nicotine lozenges. Yet, irrespective of their experiences, many acknowledged that they did not know how each NRT product worked and how to use them. Some participants expressed concerns about getting too much nicotine when they were advised to use nicotine patches along with either nicotine gum or lozenges. Several participants already had strong negative attitudes toward the medications based on their past experiences and refused to use any NRT products. They talked at length about the side effects of the medications, such as headache and stomachache, and pointed out that the medications had no effects on reducing cravings for smoking.

#### Comprehensive readiness

Many participants talked about psychological, social, and environmental changes needed to be comprehensively ready to initiate NRT. Although some started NRT without any preparation, those who seriously thought about quitting smoking prepared themselves for the use of NRT, as shown by quotes in Table 3. Many stated that this kind of preparation was needed so that they could avoid a relapse to smoking, at which they might be likely to stop using the medications. Some participants mentioned that they needed to be prepared for the dose reduction of nicotine patches as they stepped down from 21 to 14 and 7 mg. They also sought social support by telling family members or friends about their decision to quit smoking by using NRT and making public announcements at work or at social gatherings that they were quitting smoking. In addition, they talked about how they prepared their surroundings for the first day of using NRT. Some mentioned preparing the environment was crucial because it could help them avoid various temptations to smoke. For example, some picked the day when they visited a family member or a friend who lived in a smoke-free housing complex.

#### Experiential learning

A good number of participants pointed out challenges in using NRT. They talked about several occasions, such as after taking a shower or after swimming, when they forgot to put their patch back on. They said that this forgetting tended to happen as they experienced few cravings for smoking and felt comfortable with their new daily routines without smoking. Some talked about challenges in wearing a nicotine patch in hot weather because of sweating. After having used NRT for several weeks, many shared that using NRT involved learning from trials and errors. They learned how to manage cravings and how much commitment they needed to adhere to NRT. They also learned that they could override cravings by adhering to NRT. They gradually learned how to manage various challenges and make progress, which eventually helped them gain confidence and be committed to the treatment.

### Integration of Quantitative and Qualitative Results

The qualitative findings were mainly congruent with the quantitative results (Table 4). Of the 38 participants whose statements were analyzed, 34 (89.5%) showed concordances between baseline attitude scores and statements about NRT, while the remaining four participants (10.5%) presented discordance. Participants with more negative attitudes were significantly less likely to adhere to NRT (Table 2). Based on qualitative data, those with high positive attitudes were likely to adhere to NRT. However, the quantitative data failed to show statistical significance. Among the four individuals who showed discordance, one participant, who showed more positive than negative attitudes toward NRT at baseline but did not adhere to the treatment, said the patches received from the study team were not the same as those she used before. She commented that the patches did not stay on her skin, which made her stop using them. Some others stated that they were surprised to learn that NRT was not strong enough to take away cravings for smoking. Accordingly, their statements reflected these negative experiences, which differed from their attitudes toward the treatment at baseline. On the other hand, none who had more negative attitudes than positive ones at baseline changed their stance after the treatment.

**Table 4:** Joint Display of Patient Experiences With Nicotine Replacement Therapy

Type	Gender/age	Advantages subscale scores	Drawbacks subscale scores	Adherence to NRT^a^	Participant’s narratives
Concordant	M/60	3.75 >	0	Yes	I did quit smoking for 5 years. I did it with nicotine gum and Zyban.
	F/55	3.13 >	1.25	Yes	When I have my patch on, I haven’t had much desire [for a cigarette] … I haven’t had any negative effects from it at all.
	F/64	3.13 >	0.75	Yes	When I started using them [nicotine patches and lozenges] this week. I have been smoke-free for two days. I think I stopped smoking since Sunday, and I don’t even think about it. I still use lozenges regularly.
	F/66	1.75 <	3.0	No	My experience before was bad with patches. The gum was like a crap. It had a nasty taste.
	M/62	2.14 <	2.25	No	The patch made me uncomfortable. So, I didn’t sleep well. That’s why I didn’t wear it. Another challenge was to stay asleep. I think I don’t need it [the patch] anymore.
Discordant	F/57	3.5 >	0.75	No	There are a couple of nights I tried to put the patch on, and I went to bed, and I was so nauseous the next day. I am not sure what it was because of the patches or if I’ve got food poisoning. I removed the patch then. I have not tried the lozenges yet because after the patch, I was so nauseous. I felt sick.
	F/56	3.43 >	0	No	The brown patches for some reason, they don’t stick to my skin. They don’t work for me. I don’t know why. It won’t. Certain ones work, certain ones don’t.

^a^Nicotine replacement therapy.

## Discussion

To the best of our knowledge, this is the first study comparing participants’ baseline attitudes toward NRT with their statements about the treatment during counseling sessions for smoking cessation. Findings from the quantitative data are congruent with the current literature, namely, those with more depressive symptoms [24, 34] and those with more negative attitudes toward NRT [12] adhere less to the treatment than their respective counterparts. On the other hand, positive attitudes were not a significant predictor. Etter and Perneger reported a longer duration of NRT use among individuals with more positive and less negative attitudes [12]. The lack of a significant finding with positive attitudes in the present study might be related to its small sample size. None of the sociodemographic factors, including age, gender, and education, was a significant predictor, which might be related to either the small sample size or the characteristics of participants in the present study. Participants were restricted to those aged 55–77 years who were eligible for lung cancer screening, which caused a lack of variety in ages. In addition, most participants were females and had postsecondary education.

Depressive symptoms were found to be a significant barrier to NRT adherence. The low energy and fatigue that individuals with depression experience may make it a challenge to adhere to NRT dosing schedules. Ojo-Fati et al. [18] indeed found that homeless people who were depressed at baseline had low confidence to quit, were less motivated to quit, and were less likely to adhere to NRT. There is a plethora of studies reporting baseline depressed symptoms as a significant predictor of post-quit nicotine withdrawal symptoms [34, 35]. However, it is not clear whether people who are depressed are more likely to have withdrawal symptoms because of not adhering to NRT or because of severe withdrawal symptoms despite NRT use. More studies are needed to explore the underlying mechanism between depression, nicotine withdrawal symptoms, and NRT adherence.

The thematic analysis of participants’ statements revealed that NRT adherence is a learning process composed of three distinctive but interrelated phases: information needs, comprehensive readiness, and experiential learning. In the first phase, before starting NRT, many inquired about the safety of each NRT product, how to use the product, and its therapeutic effect. These findings were consistent with those from previous studies reporting expectations of NRT effect and safety concerns as major themes in relation to NRT adherence [36, 37]. Although we provided written information materials, participants might have wanted to hear directly from counselors ensuring the effectiveness and safety of the products. Similarly, engaging with information and support was identified as one of the key components for optimal NRT use [38]. The reassurance helped them be more mentally prepared for NRT use and motivated to start the medications. In the second phase of comprehensive readiness, participants prepared their physical and social environments before the initiation of NRT use. Their desire for social support seems to be identical to the component of social opportunity in the COM-B model by Mersha et al. [11]. According to them, this component consists of factors outside of the individual that make the performance of the behavior possible. The third phase of experiential learning shows the psychological and physical capabilities of the COM-B model. Participants’ experiential learning helped them gain the knowledge and skills for managing nicotine withdrawal symptoms.

Most participants’ statements about NRT were congruent with their score on the ANRT-12. Since it is less time and labor intensive to administer the scale, it can be used as a screening tool, especially in population-level programs like Quitline services. It can be used to identify candidates who are more likely to have issues adhering to NRT. For individuals with more negative attitudes, counselors can devote more attention to understanding why they would harbor negative attitudes and how to provide more information or reassurance. For example, if an individual has any side effects in earlier attempts, information on how to manage the side effects or on alternative options may allay some of their fears. Although it was modified on April 2, 2013, the earlier labeling that instructed against concomitant use of an NRT product with any other nicotine-containing medications, including cigarettes [39], made many people fear the possibility of nicotine overdose if using NRT simultaneously with another nicotine-containing product or while smoking. Again, more information on how NRT works and reassurance about conditions of safe usage may empower these people to try and remain adherent to NRT.

As in most addiction recovery processes, the first step of smoking cessation is the individual’s self-realization of the harm and the willingness to give up smoking. Behavioral, pharmacological, and other forms of support can be administered to help individuals quit smoking and are likely helpful when they are motivated to quit smoking. Similarly, NRT, an important tool for helping people with nicotine dependence, can be effectively administered if the individual is open to trying it out. The insignificant relationship between positive and negative attitudes toward NRT found in this study indicates that despite being positive toward using NRT, participants may still harbor some negative attitudes, which may hinder their sustained adherence to NRT. Although motivational interviewing techniques have been widely used to help unmotivated individuals quit smoking, the latest Cochrane Review suggested insufficient evidence of the intervention [40]. Future studies should explore whether motivational interviewing techniques could confer benefits for unmotivated individuals in NRT adherence. Another option is to use health communication messages to enhance interest and motivation to use NRT [41, 42]. Tailored and targeted health communication messages have been found to be a powerful tool to enhance cessation efforts. However, few studies have been focused on using messages to increase adherence to NRT.

### Study Limitations

There are several noteworthy limitations to mention. First, the sample size, especially male participants, was too small to identify possible covariates of NRT adherence. Second, participants were those who signed up for a smoking cessation intervention that required a time commitment. They may differ in their attitudes toward NRT from those who currently smoke but are not interested in quitting. Since participants in a clinical trial are eager to join a smoking cessation intervention, there may be a positivity bias in filling out the ANRT-12. Third, most participants were older White women in the ages of 50s and 60s. Therefore, the findings may not be generalizable to other age groups, racial and ethnic groups, and men. Replicating the study at a population level will enable researchers to have enough men and people of color for subgroup analyses. Fourth, the correlational nature of the variables in the multivariable analysis might have caused the nonsignificant finding of anxiety symptoms. Fifth, readers should also be cautious in making causal conclusions about the relationships between negative attitudes toward NRT and NRT adherence and between depressive symptoms and NRT adherence. Sixth, we did not transcribe all participants’ audiotaped counseling sessions. We purposively sampled participants as most qualitative studies adopt this methodology in sampling.

## Conclusion

NRT, paired with behavioral therapy, is one of the most effective tobacco dependence treatments [4, 5]. Our mixed-methods approach provides insight into the usefulness of measuring attitudes toward using NRT. The ANRT-12, specifically the drawbacks subscale, predicted actual adherence to NRT. The high degree of concordance with participants’ statements validates using the ANRT-12 as a potential screening tool. The ability to identify individuals with negative attitudes toward NRT may help counselors proactively address their concerns, which may increase their adherence to the treatment and, ultimately, their ability to quit smoking.

## Supplementary Material

kaae006_suppl_Supplementary_Tables_1

## Data Availability

The protocol, informed consent, data dictionary, and code book will be made accessible in data repositories. Patient, clinical and questionnaire data will be collected in an electronic data capture system (REDCap) and analyzed using SAS statistical packages. Interview data will be deidentified and transcripts will be available. Relevant resources such as code used for data processing and analyses will be made publicly available through GitHub (http://github.com). Data will be submitted to DataVerse, which is the Harvard University data repository, and variable level metadata will be provided and will include details of common data elements, definitions, and standards used for data collection and sharing as applicable.
