# Supplementary material for: Predictors of Nicotine Replacement Therapy Adherence: Mixed-Methods Research With a Convergent Parallel Design
Source: Ann Behav Med. 2024 Feb 24;58(4):275–85. doi: 10.1093/abm/kaae006 (PMC10928836; doi:10.1093/abm/kaae006)
Supplement: kaae006_suppl_Supplementary_Tables_1 [file kaae006_suppl_supplementary_tables_1.docx]

Supplemental Table 1

*Correlation coefficients among key variables*

| Variable | 1 | 2 | 3 | 4 | 5 | 6 | 7 | 8 | 9 | 10 |
| --- | --- | --- | --- | --- | --- | --- | --- | --- | --- | --- |
| 1. Age |  |  |  |  |  |  |  |  |  |  |
| 2. Education | 0.119 |  |  |  |  |  |  |  |  |  |
| 3. Alcohol Use | 0.076 | 0.164 |  |  |  |  |  |  |  |  |
| 4. Years of smoking | 0.518*** | -0.034 | -0.030 |  |  |  |  |  |  |  |
| 5. Cigarettes per day | -0.041 | -0.077 | -0.231* | 0.125 |  |  |  |  |  |  |
| 6. Depressive symptoms | -0.168 | -0.147 | -0.188 | -0.005 | 0.114 |  |  |  |  |  |
| 7. Anxiety symptoms | -0.209 | -0.115 | 0.077 | -0.023 | 0.123 | 0.609*** |  |  |  |  |
| 8. Positive attitudes | -0.060 | 0.060 | 0.088 | 0.014 | 0.021 | 0.016 | 0.111 |  |  |  |
| 9. Negative attitudes | 0.170 | -0.141 | 0.137 | -0.058 | 0.071 | -0.056 | 0.007 | -0.095 |  |  |
| 10. Weeks of NRT use | 0.084 | 0.204 | -0.058 | 0.177 | -0.015 | -0.313** | -0.227 | 0.194 | -0.378** |  |

Note. *p≤0.05, ** p≤0.01, ***p≤0.001
